# Relationship between diarrhea and *Clostridium difficile*

**DOI:** 10.1186/1471-2334-13-S1-P48

**Published:** 2013-12-16

**Authors:** Ruxandra Laza, Alexandru Crişan, Emilia Nicoară, Narcisa Nicolescu, Sorina Laitin, Oana Plavițu, Karina Bota

**Affiliations:** 1 Victor Babeş University of Medicine and Pharmacy, Timişoara, Romania; 2 Dr. Victor Babeş Clinical Hospital of Infectious Diseases and Pneumology, Timişoara, Romania

## Background

With the widespread use of antibiotics, *Clostridium difficile* bacillus, normal resident of the colon, became more frequently involved in the production of pseudomembranous colitis.

The fact that in the Clinic of Infection Diseases II Timişoara we have registered many cases in the last 8 month, led to the following questions: a) has the disease occurred in the context of a particular field or therapeutic act (interventions on the digestive tract); b) were there flaws in the oversight of the hygiene state; c) are we witnessing a process of infection “pathomorphosis”; d) is the only evidence of additional diagnostic efficiency measure.

## Methods

*Clostridium difficile* was found to be involved in triggering diarrheal syndrome in 32 patients hospitalized in the period January-August 2013; the authors aim to analyze the possible presence of favorable conditions in which bacteria may increase aggression. The following elements have been analyzed: age, sex, comorbidities, presence and duration of antibiotic therapy prior to the onset of diarrheal disease, the origin of the case (from the hospital or family), data on disease evolution.

## Results

*Clostridium* etiology represented 44% of all cases of hospitalized diarrhea in the mentioned range (94 cases). 62% of the patients analyzed were aged over 60. In patients with diarrhea caused by *C difficile* multiple comorbidities have been reported in 87.5%, malignancies in 18.75%. Antibiotic therapy was present in 87.5% of cases. 65% of patients had undergone surgery (digestive, uro-genital, cardiovascular, osteoarticular). The evolution was favorable in 62% of patients, 25% had recurrence, death was reported in 12% of cases. The disease was linked with hospital contact in 97% of cases.

## Conclusion

A high percentage of cases with diarrhea caused by *C difficile* were noticed in immunocompromised patients. In patients without significant pathological past, the first interpretation seems to be nosocomial character. The diseases that have been reported in patients without pathological past, draw attention on the pathomorphosis process, amending the etiology, epidemiology and clinical infectious diseases profile.

